# Enhancing Sensor Network Security with Improved Internal Hardware Design

**DOI:** 10.3390/s19081752

**Published:** 2019-04-12

**Authors:** Weizheng Wang, Zhuo Deng, Jin Wang

**Affiliations:** 1 School of Computer & Communication Engineering, Changsha University of Science & Technology, Changsha 410114, China; peakexpe@csust.edu.cn (W.W.); dz5019@stu.csust.edu.cn (Z.D.); 2 Hunan Provincial Key Laboratory of Intelligent Processing of Big Data on Transportation, Changsha University of Science & Technology, Changsha 410114, China; 3 School of Information Science and Engineering, Fujian University of Technology, Fuzhou 350118, China

**Keywords:** sensors, Internet-of-Things, sensor networks, information security, cryptographic chips

## Abstract

With the rapid development of the Internet-of-Things (IoT), sensors are being widely applied in industry and human life. Sensor networks based on IoT have strong Information transmission and processing capabilities. The security of sensor networks is progressively crucial. Cryptographic algorithms are widely used in sensor networks to guarantee security. Hardware implementations are preferred, since software implementations offer lower throughout and require more computational resources. Cryptographic chips should be tested in a manufacturing process and in the field to ensure their quality. As a widely used design-for-testability (DFT) technique, scan design can enhance the testability of the chips by improving the controllability and observability of the internal flip-flops. However, it may become a backdoor to leaking sensitive information related to the cipher key, and thus, threaten the security of a cryptographic chip. In this paper, a secure scan test architecture was proposed to resist scan-based noninvasive attacks on cryptographic chips with boundary scan design. Firstly, the proposed DFT architecture provides the scan chain reset mechanism by gating a mode-switching detection signal into reset input of scan cells. The contents of scan chains will be erased when the working mode is switched between test mode and functional mode, and thus, it can deter mode-switching based noninvasive attacks. Secondly, loading the secret key into scan chains of cryptographic chips is prohibited in the test mode. As a result, the test-mode-only scan attack can also be thwarted. On the other hand, shift operation under functional mode is disabled to overcome scan attack in the functional mode. The proposed secure scheme ensures the security of cryptographic chips for sensor networks with extremely low area penalty.

## 1. Introduction

In recent years, Internet-of-Things (IoT) has developed rapidly; it connects various objects around the world through the internet. By combining with IoT, sensors play a more powerful role and are benefiting greatly the human beings [1,2,3,4,5]. Currently, sensor networks based on IoT are being extensively used in smart cities, health care, intelligent transportation, industrial monitoring, etc. [6,7].

With the rapid growth of sensor networks, the information security and privacy become main concerns and challenges [8,9]. Hence, security management for sensors networks becomes more and more important. Cryptographic algorithms are commonly used to ensure the sensor networks data security [10,11]. They can be divided into symmetric-key cryptographic algorithms, such as DES (Data Encryption Standard) and AES (Advanced Encryption Standard), and asymmetric-key ones, such as ECC (Elliptic Curves Cryptography) and RSA (Rivet Shamir Adelman). Symmetric-key cryptographic algorithms utilize the same cipher key in the process of encryption and decryption. In contrast, asymmetric-key cryptographic algorithms utilize two cipher keys, i.e., a public and a private key for encryption and decryption respectively.

To achieve the acceptable throughput and reduce computational resource requirements, cryptographic algorithms are usually implemented in specific hardware [12]. The symmetric-key cryptography—AES algorithm is regarded as the most appropriate algorithm for sensor networks as its hardware implementation has performance advantages, e.g., lower chip area and higher throughput [13]. In symmetric-key cryptography, both encryption algorithm and decryption algorithm are open to the public, but there is no hope of cracking the cipher key using known plaintext and ciphertext pairs. The private key is generally stored inside the non-volatile memory of crypto chip and prohibited from accessing easily by users. However, the security of cryptographic system may be threatened if the cipher key is accessed and deduced in an oblique and sophisticated manner.

As the accuracy of cryptographic algorithm is highly demanding, the crypto chip should be rigorously tested to guarantee it can properly operate. Scan design is the most widely used structured DFT technique in industry, which brings great convenience to production testing and online debugging. Such DFT technology can control and observe the state of flip-flops by replacing them with scan cells, and the controllability and observability of integrated circuit (IC) is improved dramatically. As a result, automatic test pattern generation (ATPG) becomes effortless, and high fault coverage and little test application time can be achieved easily [14,15,16]. Nevertheless, scan design opens out a backdoor for illegal user to steal encryption key from cryptographic chip. The security of cryptographic hardware is threatened severely by the scan-based noninvasive attack. After encryption algorithm is implemented in cryptographic chip for one round during functional mode, the intermediate encryption results are stored in scan chains. If permitted, at this time the adversary may switch the circuit into test mode to shift out the intermediate states by scan operation and observe at the output ports of scan chains. It is probable to derive the encryption key by using a certain number of pairs of plaintext and intermediate state. Scan based attack is easier to execute and poses more serious potential menace to cryptographic circuit than those based on side-channel parameters, such as timing analysis, power consumption and electromagnetic radiation [17]. The hardware security problem can’t be ignored even for the purpose of testability. At the same time, it is not inadvisable to compromise the testability for security by discarding the scan-based DFT technique. Therefore, the test methodology, which does not hurt the security of cryptographic chip while maintaining the desirable test efficiency and quality, should be developed urgently.

The scan-based side-channel attack was firstly presented by Yang et al. in [18]. They stated that the adversary could employ differential cryptanalysis base on the readout intermediate values to deduce the private key of a DES chip. It has been reported that crypto systems implementing cryptographic algorithms such as ECC, RSA, and AES are also vulnerable to scan-based side-channel attack [19,20,21]. These scan-based side-channel attacks are under assumption that the values of scan chains could be accessed by converting circuits from functional mode to test mode [19,22]. The adversary first resets the chip to an initial state. Then he applies the pre-calculated test vectors (i.e., plaintexts) to cipher module and capture intermediate encrypted result into scan chains in functional mode. The chip is subsequently switched into test mode and these intermediate values are scanned out for analysis through the output pins of scan chains. Such attacks are described as mode-switching attacks. The drawback of these attacks is that the whole process requires both two work patterns: functional mode and test mode.

Test-mode-only attacks [23,24,25] that can be implemented only under the test mode are deemed to be more risky attacks. Such attacks mainly focus on boundary scan design, in which each primary input (PI) is equipped with a boundary scan cell. At the beginning of a cracking cycle, the cryptographic chip is initialized to one certain state. Then the pre-computed plaintext is delivered in boundary scan chain under the shift phase of test mode. Next, the crypto chip enters capture phase of test mode. In the meantime, encryption is conducted for one cycle and the encrypted result is stored in scan chains. Afterwards, the crypto chip enters shift phase of test mode again. The intermediate encrypted state outflows via the outputs of the scan chains for cryptanalysis. In test-mode-only attacks it is not necessary that the crypto circuit has to jump into the functional mode for loading the plaintext. Thus, test-mode-only attacks are resistant to countermeasures against mode-switching attacks.

Advanced DFT architecture such as on-chip decompressor, on-chip compactor and X-masker [26,27], used to be considered as natural defense against scan based side channel attacks [28]. Recently such architecture has been proved not unassailable [29,30]. Enhancing the security of a chip has become one of main concerns to industry, and a variety of countermeasures have been developed, mainly including the following five categories:

1. Protection of mode switching: By introducing the test controller, the values of the scan flip-flops will be reset once switch from functional mode to test mode is requested [31,32,33]. However, this countermeasure is solely applicable for mode-switching attacks and powerless to test-mode-only attacks.
2. Blocking of encryption key: In reference [22], secure scan architecture including MKRs (Mirror Key Registers) is utilized. it exploits two operation modes: insecure and secure modes. In insecure mode, the cipher key is prohibited from entering MKRs, but test vectors can be loaded into scan chains and test responses can also be captured and scanned out. In secure mode, the cryptographic circuit can work properly but can not return to insecure mode to conduct test and debug operation. For this type of countermeasure, it may be impossible to gain encryption data corresponding to a known plaintext discretionarily. However, the clunky test control architecture brings negative impact on IP design.
3. Improvment of scan architecture: In reference [34], a secure scan architecture called differential scan path is proposed to improve the chip security. In the technique, the state of the scan path is divided into two segments. In test mode, only subtraction of the segment states can be observed at the scan-out ports. Deriving the intermediate state from the difference results needs much guesswork. The guessing probability decreases exponentially when the length of the scan path increases.
4. Obfuscation of scan-out data: This countermeasure inserts obfuscation logics such as dummy flip-flops, exclusive-or (XOR) gates, inverters, lock and key logic, into the scan chains to change scan-out data randomly [35,36,37,38,39,40]. If the scan-out data is obfuscated, an attacker may be misguided to deduce the inaccurate key or be unable to calculate the cipher key. The scan-out encryption result is obfuscated by dynamically altering the join order of the sub-chains in [35,36]. Nevertheless, the calculating signature attacks can still be implemented even if attackers don’t know the scan architecture or scan flip-flop order [41,42]. A key and lock method was introduced in [37] to thwart signature attacks. Several scan flip-flops are selected to make their shift-enabling signal controlled by the values of an additional shift register (i.e., test key). In test phase, the reshuffled scan cells controlled by inaccurate bits of test key will remain in functional mode instead of test mode. Obfuscation of scan data is achieved as the scan-out data is actually not the test response captured in scan chains. To resist test-mode-only signature attack, an improved technique refereed as dynamic obfuscation of scan data was also presented in [37]. The inaccurate test key is cyclically shifted in test phase, and thus the scan-out data will be rather more erratic. However, this modified scan design involves some hardware overhead and cannot apply to delay test based on Launch-off-Capture (LOC).
5. Scan Chain Encryption: The secure technique proposed in [43] uses the secret-key management policy to encrypt the scan chain content during test. Only the test engineers who have the key can deliver desired data into scan chains and shift out intermediate states from scan chains. The secure scheme proposed in [44] encrypts the data written to or read from the scan chains by using an on-chip lightweight block cipher. Such techniques require a crypto core insertion in the scan chains, which increases the complexity of scan design.

In this paper, we propose a secure scan scheme to protect cryptographic chips with symmetric key in sensor networks against scan-based attacks. To resist mode-switching attack, the proposed scheme uses a mode-switching detection (MSD) signal to take over the control of system reset signal for scan cells. The MSD signal is achieved by Xor previous working mode with current working mode. Once the mode switching happens, the reset input of scan cells is set to high level, which can initialize the system. In the proposed scheme, the round key register is configured into scan chains for ensuring the test quality of cryptographic chip. To resist test-mode-only attacks, the proposed secure technique also secludes the secret key from the encryption unit under test mode by reshuffling the control input of multiplexer connected to the data input of key register. To overcome functional-mode attack, the shift-enabling input of each scan cell is controlled by the result of the AND operation on system shift-enabling signal and working mode selection signal. This guarantees that shift operation is disabled under functional mode. Consequently, the proposed scheme can resist the scan-based side-channel attacks reported yet with extremely low area penalty and it doesn’t compromise the testability of cryptographic chip. The proposed technique is demonstrated on AES chips but can be extended to cryptographic chips with symmetric key.

## 2. Preliminaries

### 2.1. Scan Design

In a sequential circuit, multiple clock cycles must be applied to control and observe the states of flip-flops. The full-scan design adapts flip-flops to make them controlled and observed directly throughout shift operation. Accordingly, the sequential circuit testing is transformed into the combinational circuit testing by scan design. In virtue of convenience to IC testing, the scan design becomes a widely utilized DFT technique in industry today. Besides, the testing for a chip with a large number of I/O ports is one great challenge since the automatic test equipment mostly has only limited data channels. This problem is disposed of successfully by boundary scan design, which equips each chip input/output with a boundary scan cell and serially connects these boundary scan cells into a boundary scan chain [16].

**Figure 1:**
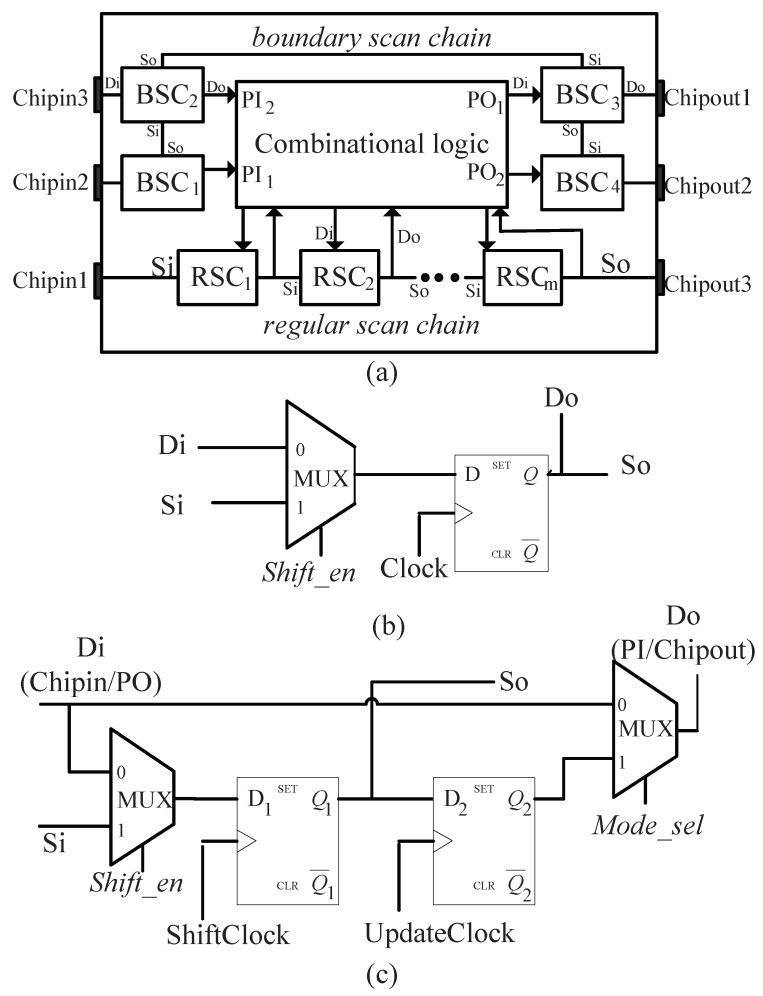
Scan design. (**a**) Scan architecture including regular and boundary scan chain. (**b**) Internal architecture of a RSC. (**c**) Internal architecture of a BSC.

The DFT scan structure including regular and boundary scan chain is shown in Figure 1a. By adding one 2-to-1 multiplexer at the input, the internal flip-flops are configured as regular scan cells (RSC), as illustrated in Figure 1b. A RSC has two alternative input sources: data input (Di) and scan input (Si). The Di is driven by the combinational logic of IC, while the Si is driven by the output of another RSC. The shift-enabling (Shift_en) signal controls which data to propagate into flip-flop. In a RSC the data output (Do) and the scan output (So), which drive the combinational logic of IC and Si of another RSC respectively, are shared. A typical boundary scan cell (BSC) consists of two D flip-flops and two multiplexers, as illustrated in Figure 1c. The BSC could be inserted at the input port or output port of the chip. As an input BSC, the input source Di is driven by a chip input (Chipin) port, and the data output Do corresponds to a primary input (PI) of the internal logic. As an output BSC, the input source Di is connected to a primary output (PO) of the internal logic, and the data output Do corresponds to a chip output (Chipout) port. Values propagated to Do are selected by the working mode selection (Mode_sel) signal. The regular/boundary scan chain is formed by tying the SO of a RSC/BSC to the SI of the succeeding RSC/BSC. A scan chain can be externally accessed by connecting the Si of the first scan cell in it to a chip input pin and the So of the last scan cell in it to a chip output pin. It is possible to shift in arbitrary values to scan chains from Si pins while shifting out the states of scan chains through So pins.

The working mode is described briefly as following:

- When the chip runs in the functional mode, Mode_sel = 0 and Shift_en = 0. The RSCs are driven by combinational logic, and BSCs are transparent (data passes from Di directly to Do).
- When the chip runs in test mode, Mode_sel = 1 and there are three operation phases: Shift, Update and Capture.
  - In the Shift phase, Shift_en is assigned to ‘1’ and clock pulses are applied to ShiftClock of each BSC and clock input of each RSC such that test patterns can be scanned in from Si of (boundary and regular) scan chains and test responses can be scanned out through So of scan chains.
  - In the Update phase, which targets only BSCs, the test data stored in D1 (termed as the capture flip-flop) are propagated to D2 (termed as the update flip-flop) by giving a clock pulse to Updateclock of each BSC. At this time, the state of D2 determines the Do of BSC.
  - In the Capture phase, Shift_en is set to ‘0’, one clock pulse is applied to ShiftClock of each BSC and clock input of each RSC, and the test response at Di will be captured into RSC or the D1 of BSC.

### 2.2. AES and Its Hardware Implementation

**Figure 2:**
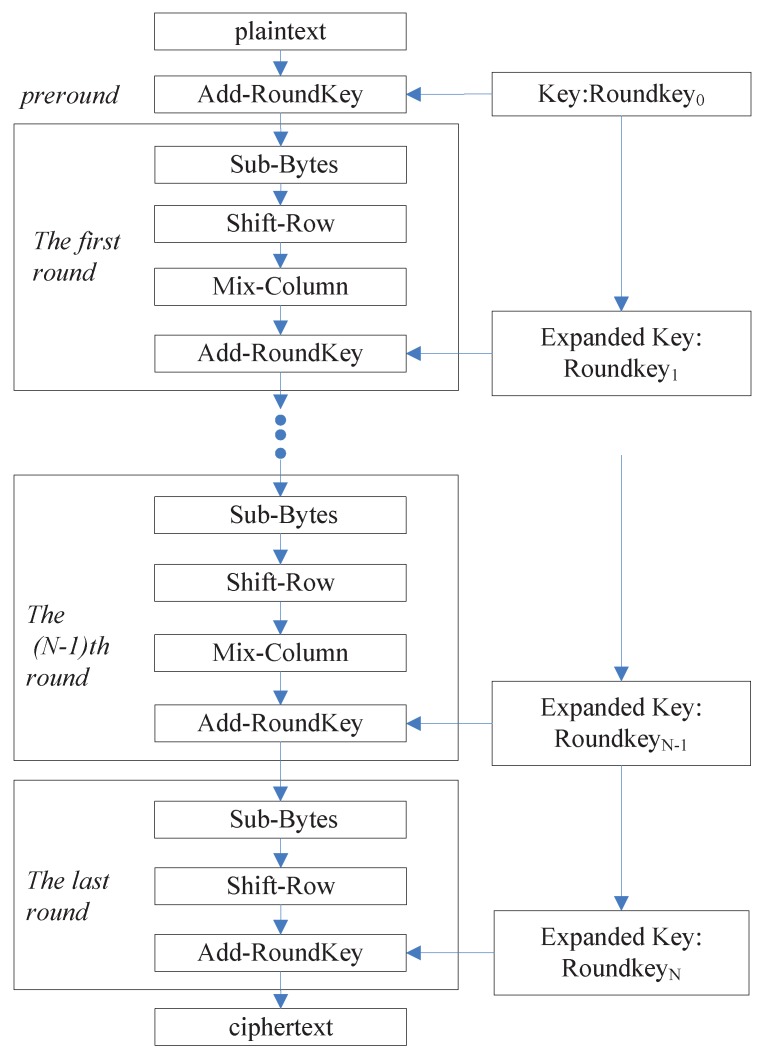
The flow of encryption of AES algorithm.

Due to high processing speed and high level of security, AES has been regarded as the symmetric-key block cipher standard and widely implemented in hardware. The AES is a 128-bit block cryptographic algorithm with three kinds of key lengths. The key length may be 128, 192, or 256 bits. The encryption process includes multiple operation rounds which relies on the key-lengths, i.e., 10 operation rounds for 128-bit key, 12 operation rounds for 192-bit key, and 14 operation rounds for 256-bit key. As illustrated in Figure 2, one round comprises 4 fundamental transformations: Sub-Bytes, Shift-Rows, Mix-Columns and Add-RoundKey, except for the last round in which Mix-Columns is not contained. In the encryption algorithm, the 128-bit input block is known as plaintext and the equal-size output block is known as ciphertext. The output result of an anterior round is known as state.

Using a substitution function called S-Box, Sub-Bytes performs a nonlinear substitution operation on each input byte. Shift-Rows rotates each row of state matrix from right to left by several bytes, according to the location of the row. Mix-Columns is the 4-byte mingling transformation among each column of the state matrix. Add-RoundKey is the exclusive-or (XOR) operation of a round key and a state. The detailed introduction on the AES can be referred to [45].

In a pipelined or iterative AES hardware, the one-round implementation consuming one clock pulse is typically utilized [45]. During the initial clock pulse, the plaintext is applied and the temporary state is stored in a state register. Generally, the encrypted state of preround is not deposited since it includes only a bitwise XOR operation. In the following rounds, the result of each round is also stored. In the iterative AES hardware module, the output of the state register, as the input of the next round, goes back to the input of AES module via a multiplexer. The ciphertext is obtained after all the round operation is carried out with different round keys. In the pipelined hardware module, the one-round architecture is reproduced 10 (12 or 14) times. The output of a round architecture drives the input of the next round architecture. The state register of the last round architecture stores and outputs the ciphertext.

## 3. Proposed Secure Scan Test Scheme

If an attacker can access the state register through scan design, the intermediate result of just one round is available for him. Thus, the cipher key (also called as user key) can be exposed through mathematical derivation based on intermediate encryption results [22,23]. There are three possible attack ways based on standard boundary scan design.

1. Functional-mode attack. This attack consists of 2 steps. In the first step, a pre-computed plaintext is delivered to the primary inputs when the crypto chip works in functional mode (Mode_sel = 0 & Shift_en = 0) for only one round of AES algorithm. The state of the one round operation is stored in the scan chains. In the second step the crypto chip remains in functional mode but shift-enabling signal Shift_en is set to ‘1’ (Mode_sel = 0 & Shift_en = 1) and the encryption result in scan chains is shifted out for analysis. Such 2-step operation is duplicated for different plaintexts until the cipher key are successfully deduced.
2. Mode-switching attack. This attack also consists of 2 steps. In the first step, a pre-computed plaintext is delivered to the primary inputs when the crypto chip works in functional mode (Mode_sel = 0 & Shift_en = 0) for only one round of AES algorithm. The first step is similar with that of functional-mode attack. The only difference is that, in the second step the crypto circuit is converted into shift phase of test mode (Mode_sel = 1 & Shift_en = 1) and then the encryption result in scan chains is shifted out.
3. Test-mode-only attack. The crypto chip runs in test mode (Mode_sel = 1) throughout this attack. This attack consists of 4 steps. In the first step, the plaintext is scanned into boundary scan cells corresponding to primary inputs under shift phase (Shift_en = 1). In the second step, the plaintext is delivered to the primary inputs from boundary scan cells in update phase. Then the chip runs under capture phase (Shift_en = 0) to store the result of the round operation in the scan chains. In the last step, the crypto chip enters again shift phase (Shift_en = 1) and the round result in scan chains is shifted out while the next plaintext is shift into. The four steps are also repeated for different plaintexts until the cipher key is successfully deduced.

A standard scan-based DFT architecture should have the following characteristics:

1. The testability and debuggability of crypto core should be guaranteed by using scan-based DFT.
2. The intermediate encrypted result saved in scan chains must not be accessed to crack the cipher key. In other words, the values that could be shifted out of scan chains must be unrelated to the cipher key or be obfuscated.

### 3.1. Proposed Secure Scan Architecture

**Figure 3:**
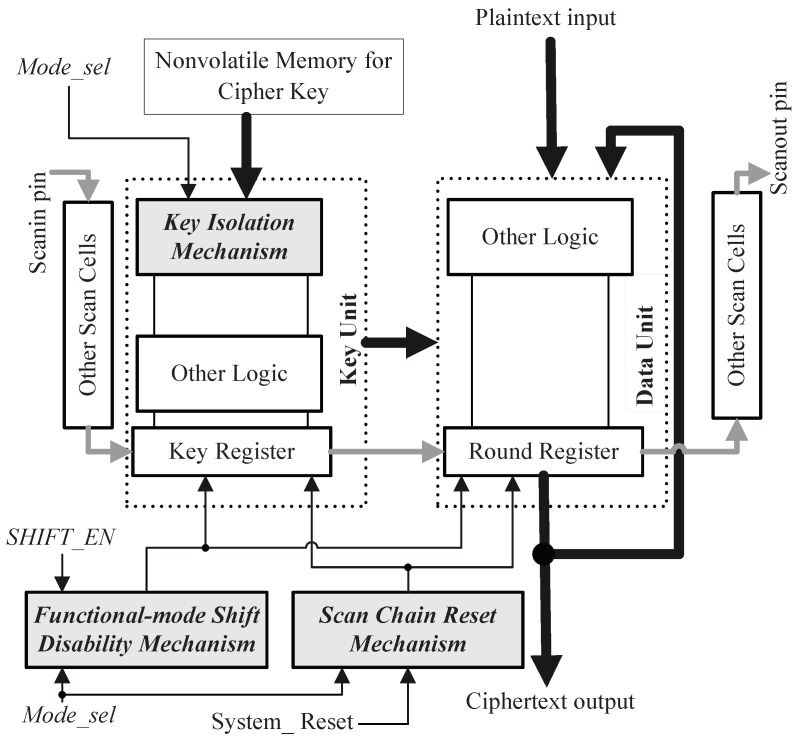
Proposed secure scan test architecture.

Based on above analysis, we present a secure scan architecture, which can protect the AES chip with boundary scan design against scan-based attack. As illustrated in [45], the original architecture of regular and scalable AES hardware contains mainly two parts: key unit which stores cipher key and calculates the round keys, and data unit which implements any AES encryption or decryption round with the round key. The presented secure scan test architecture is described in Figure 3. Besides the standard scan design, the functional-mode shift disability mechanism, key isolation mechanism and scan chain reset mechanism are added in the AES architecture. To guarantee high testability, the proposed architecture configures the key register of key unit and the round register of data unit into scan chains. The scan cells in scan chains comprise RSCs and BSCs. The working details of functional-mode shift disability mechanism, scan chain reset mechanism and key isolation mechanism are explained in detail in the following subsections.

**Figure 4:**
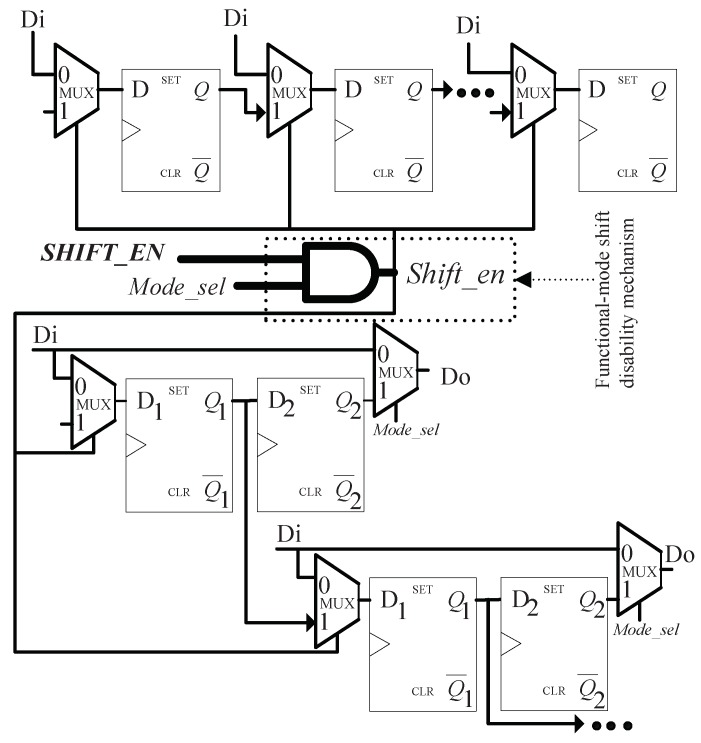
Functional-mode shift disability mechanism.

In order to prevent attackers from misusing shift operation (Shift_en = 1) to obtain intermediate encryption result in functional mode (Mode_sel = 0), the system shift-enabling pin SHIFT_EN is fed to the Shift_en port of each scan cell (including boundary scan cell and regular scan cell) via an AND gate. The other input of the AND gate is controlled by the working mode selection signal Mode_sel, as shown in Figure 4. This guarantees that Shift_en port of each scan cell can only receive ‘0’ when Mode_sel = 0, that is, the shift operation is disabled in functional mode.

**Figure 5:**
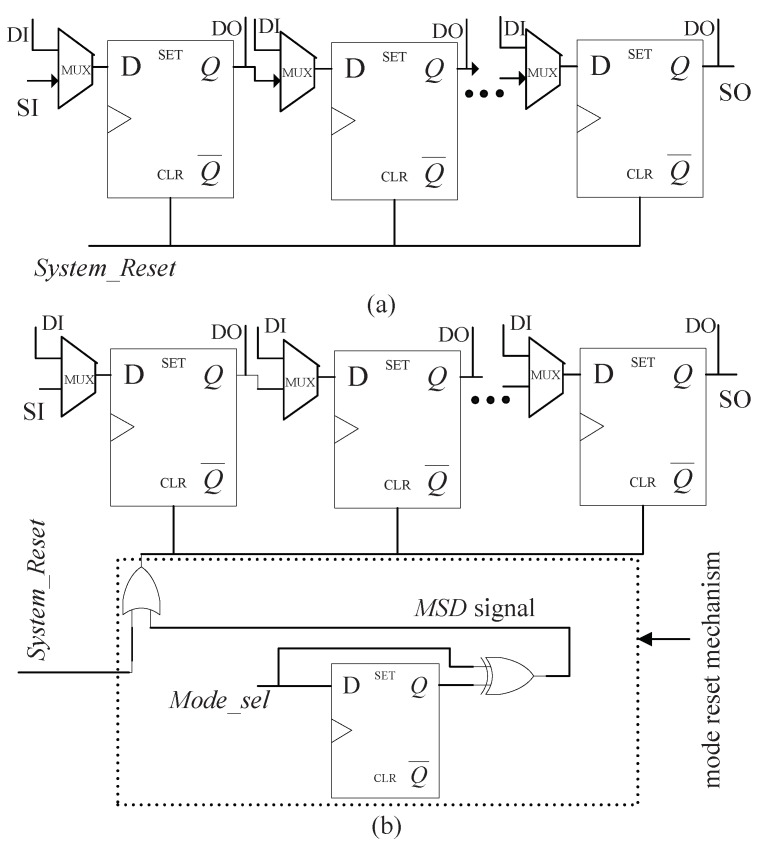
Architecture of scan chain. (**a**) Standard scan chain. (**b**) Secure scan chain with scan chain reset mechanism.

In standard scan architecture, the system reset input (System_Reset) drives solely the reset terminal (CLR) of every scan cell and is utilized to initialize the chip, as shown in Figure 5a. To perform the reset operation at the appearance of mode switching, the proposed secure scan architecture introduces the mode reset mechanism for system reset signal, which only comprises an OR gate, a D flip-flop and an XOR gate, as illustrated in Figure 5b. The inserted D trigger stores the Mode_sel value in previous clock pulse. The working mode selection signal Mode_sel is utilized to convert the AES circuit between the functional and test mode. The XOR gate output, which combines the Mode_sel in previous clock pulse and the Mode_sel in current clock pulse by using the Boolean XOR operator, is used as mode-switching detection (MSD) signal. The reset terminal of every scan cell is assigned to logical OR of System_Reset and MSD. Assume that, the reset operation of a scan cell is carried out as CLR terminal is logic ‘1’. As Mode_sel alters either from zero to one or from one to zero, the MSD signal becomes logic ‘1’ and the reset terminal of every scan cell will be given logic ‘1’ regardless of the system reset input. Just then the scan cells are cleared to protect encrypted information.

When System_Reset is ‘1’, the output value of OR gate remains ‘1’ which won’t be affected by MSD. Consequently, the system reset operation can be implemented normally. It should be noted that the extra logic gates including OR gate, D flip-flop and XOR gate can be placed in the source (near the input port) of System_Reset signal. Consequently, they will not increase the global routing complexity of System_Reset signal significantly.

**Figure 6:**
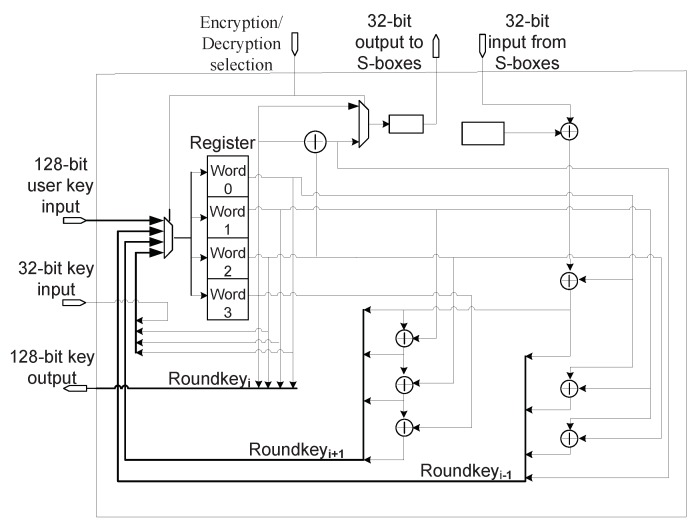
Main part of original key unit (for 128-bit AES hardware).

**Figure 7:**
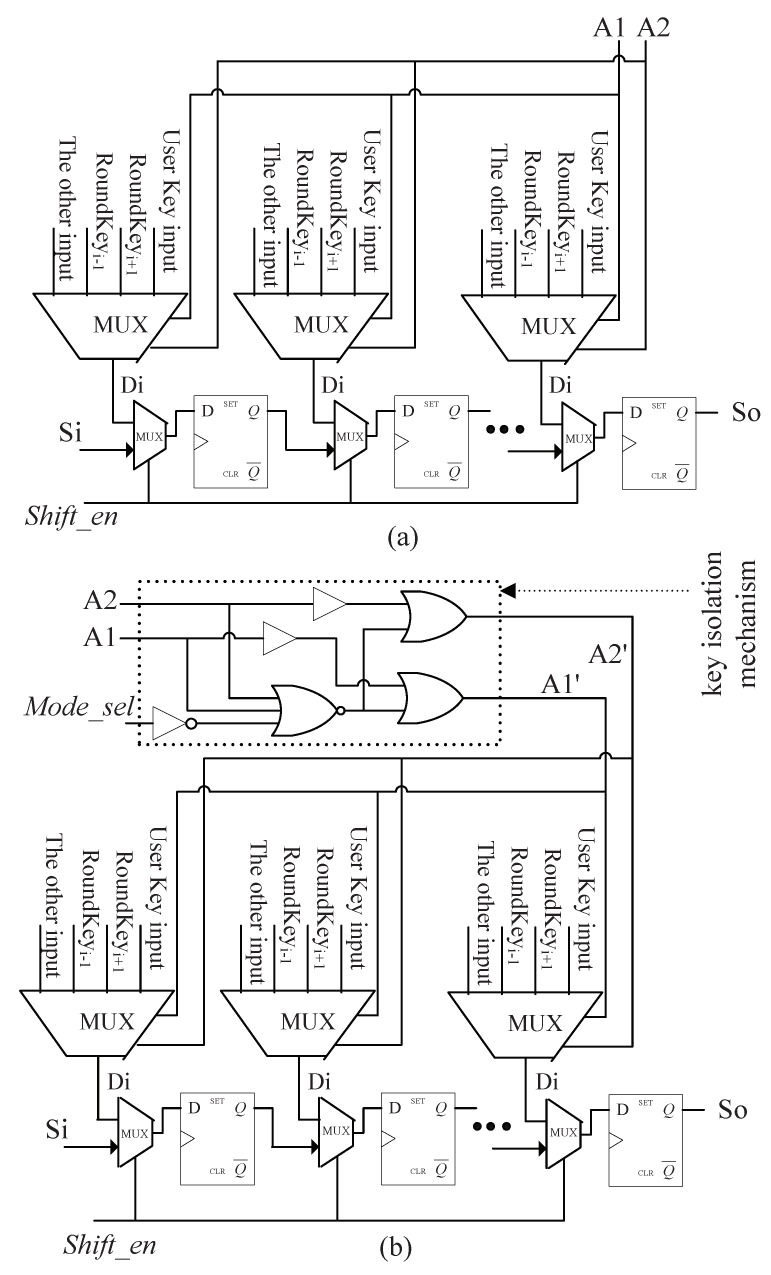
The data input and scan chain structure of key register. (**a**) the original data input and scan chain structure of key register. (**b**) the data input and scan chain structure of key register for the proposed technique.

The key isolation mechanism is inserted into key unit. The typical structure of the key unit for 128-bit AES hardware is illustrated in Figure 6, which consists of key register and other combinational logic [45]. The key register is used to store and output the generated round keys. Key units for other key sizes are similar to it. In the standard scan design, the Di of each scan cell in the key register receives four input signals via a 4-to-1 multiplexer: the user key input (Roundkey${}_{0}$), the previous round key input (Roundkey${}_{i-1}$) used for decryption, the next round key input (Roundkey${}_{i+1}$), and the other key input as described in Figure 7a. It should be noted that the user key is also denoted by Roundkey${}_{0}$. A multiplexer has two address inputs (labeled as A1 and A2 in Figure 7) that determine which data input is selected. The inputs A1 and A2 are actually the Encryption/Decryption selection signals shown in Figure 6. Supposed that, when {A1, A2} are {‘0’, ‘0’}, {‘0’, ‘1’}, {‘1’, ‘0’}, and{‘1’, ‘1’}, Roundkey${}_{0}$, Roundkey${}_{i-1}$, Roundkey${}_{i+1}$, and the other key input are selected, respectively. The cipher key is generally stored in nonvolatile memory. As the encryption circuit is switched-on and {Mode_sel, Shift_en} are set to {‘0’, ‘0’}, the chip enters the functional mode. Firstly, {A1, A2} are set to {‘0’, ‘0’}, the cipher key is delivered into the key register. In the following clock cycles, if {Mode_sel, Shift_en} remain {‘0’, ‘0’}, and {A1, A2} = {‘1’, ‘0’}, the key generator will generate and deposit the round keys (Roundkey${}_{0}$ to the last round key Roundkey${}_{N}$) using key expansion function. When doing decryption, {A1, A2} = {‘0’, ‘1’}, the round keys are generated and deposited in reverse order.

If it is allowed to load the user key into key register during the capture operation of test mode (i.e., {Mode_sel, Shift_en} = {‘1’, ‘0’}), the crypto chip will be under threat as the secret information goes into scan chains and can be shifted out from scan output pin. The key isolation mechanism makes the user key input disabled in the capture phase by modifying the address inputs of the multiplexer as shown in Figure 7b. Mode_sel is used to assist to control the multiplexer via one NOT gate, one NOR gate and two OR gates. As the crypto circuit enters functional mode (Mode_sel = 0), the added logic gates play no role, i.e., {A1, A2} are equal to {A1’, A2’}. When Mode_sel = 1, the case that {A1’, A2’} = {‘0’, ‘0’} would never happen even if {A1, A2} = {‘0’, ‘0’}. In other words, in test mode delivering the user key to key register is blocked. The relationship between {A1’, A2’} and {Mode_sel, A1, A2} is described in Table 1. It’s important to note that data from input Roundkey${}_{i-1}$ and input Roundkey${}_{i+1}$ are not real round keys if the user key is masked. They are irrelevant to the user key as they may be determined by the initial state of the key register or the test data shifted into scan cells located in key register. If the key register does not contain the user key information, the round register in data unit does not store the real any intermediate encryption result. Consequently, it’s impossible to deduce the correct cipher key from observing the scan-out data.

The two buffers inserted between A1/A2 and the OR gates are used to balance the signal propagation delay. Assume Mode_sel = 1. Without the buffers, (A1’, A2’) may produce the transition “11” -> “00” -> “11” when switching (A1, A2) from “11” to “00”. The temporal state “00” of (A1’, A2’) may bring the risk of direct disclosure of the user key. The hazardous state can be eliminated by inserting the buffers.

### 3.2. State Diagram of Proposed Secure Architecture

**Figure 8:**
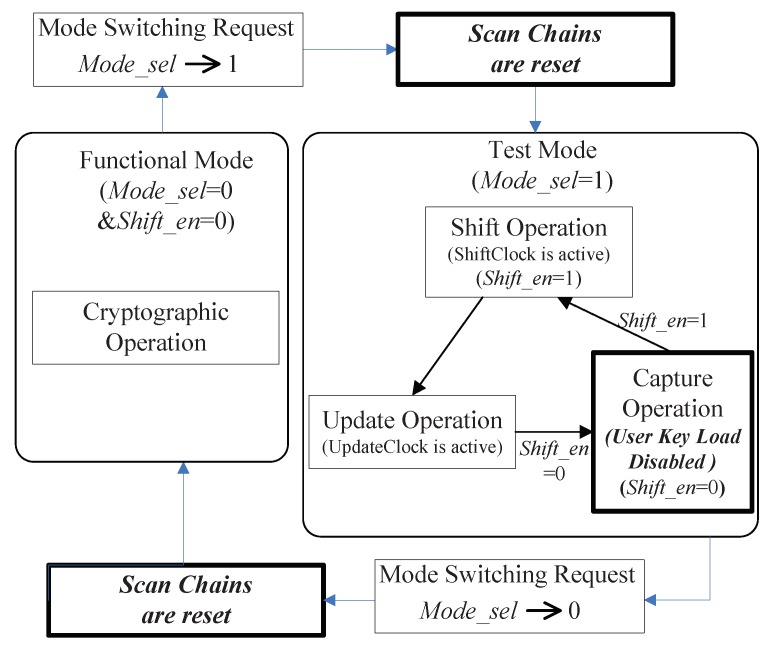
State diagram of proposed secure scan test scheme.

In the presented secure scan scheme, there are two pivotal attributes: resetting scan chains while mode switching and isolating the user key from encryption module in test mode. The state transition graph of the presented scheme is illustrated in Figure 8.

When Mode_sel is set to ‘0’ at power-on, Shift_en is also limited to ‘0’. The crypto chip is running in the functional mode. During this time, the user key can be loaded into key register and the encryption/decryption is performed.

Once Mode_sel goes from ‘0’ to ‘1’, the chip is immediately reset, namely the state of scan cells is cleared. The reset action erases the secret information stored in scan chains. Then the crypto chip works in the test mode, which consists of 2 operation phases: shift phase and capture phase. The shift-enabling input (Shift_en) is utilized to convert the circuit between these two operation phases. In the shift phase, Mode_sel = 1 and Shift_en = 1. In this period, a certain number of clock pulses are applied to ShiftClock of each BSC and clock input of each RSC such that a test vector can be scanned serially into the scan chains via the scan-input pins. Meanwhile, the previous test response is shifted out via scan-output pins. Once the test vector is scanned completely into the scan chains, one clock pulse is applied to Updateclock of each BSC. In the Update phase, the test data stored in D1 (capture flip-flop of BSC) are propagated to D2 (update flip-flop of BSC) while the test vector is applied the combinational logic through Do of scan cells. Next, Shift_en is assigned to zero to bring the circuit into capture phase for a clock cycle. In this clock cycle, the test response is loaded into the scan chains through Di. Since loading key under capture phase is prohibited in the propose scheme, it’s impossible that attackers misuse the capture phase to read the user key into key register. This guarantees that the values shifted out from scan chains are irrelevant to the cipher key.

By setting Shift_en to ‘1’ again, the test response loaded previously is scanned out of scan chains via the scan-output ports and simultaneously the next test vector is shifted into the scan chains. The crypto chip can switch its working mode freely without risk. Once the mode-switching requirement is submitted, the chip is promptly reset.

## 4. Performance Analysis

### 4.1. Testability Analysis

In the presented secure scan scheme, test vectors can be delivered into the circuit under test exactly as they do in the standard scan design. It can be used to exercise all kinds of test set, such as stuck-at test set and LOC or LOS (Launch-off-Shift) delay test set.

In order to verify the testability, the proposed secure scan scheme is conducted on pipelined [46] and iterative AES core [47] having key scheduling. The netlists of the original implementations are obtained by synthesizing with Synopsys DC (Design Compiler). Scan chains including boundary scan chain and regular scan chain are inserted into netlists with Synopsys Test Compiler. Then the proposed technique is also introduced into the netlists and synthesized by Synopsys Test Compiler. Table 2 gives the test simulation results including number of test vectors and coverage for AES cores with standard scan design insertion and proposed secure architecture insertion. The targeted fault model is stuck-at fault. The second and fourth columns show the number of test vectors obtained by ATPG for standard scan insertion and proposed secure scan insertion, respectively. The third and fifth columns show the fault coverage achieved by ATPG for standard scan insertion and proposed secure scan insertion, respectively. The change percentage of test vectors and fault coverage with respect to the standard scan scheme is also listed for the proposed architecture. For the two AES circuits, fault coverage decreases only very slightly. The major reason for slight coverage loss is that, a few faults on the transmission line transmitting the cipher key from non-volatile memory to key generator can not be tested by the proposed scan scheme. It should be noted that, such faults can be tested by simply applying the functional test for some clock cycles. The number of the required test vectors also increases within an acceptable range. Therefore, the testability of AES circuits is unaffected obviously and production test can run on the rails.

When the crypto chip runs in the functional mode, the inserted gates in the proposed architecture are transparent and thus the function of crypto chip keeps unchanged.

### 4.2. Security Analysis

Since shift operation is disabled in functional mode, attackers can not shift out round encryption result in the mode. The noninvasive attack in only functional mode can be resisted.

No matter what method the scan-based side-channel attacks use, the intermediate encryption state available from scan chains is requisite. Nevertheless, for the proposed scheme, all the encrypted results stored temporarily in scan chains will be erased in case of mode switching. Thus, the mode-switching attacks could be thwarted.

In addition, the sensitive data related to user key will never be included in scan chains in test mode because reading user key into AES key unit is invariably prevented. The round keys used in test mode are irrelevant with user key and simply depend on test stimulus shifted into scan chains or initial state of the crypto system. Hence, the proposed secure scan scheme can overcome the test-mode-only attacks as well.

This proposed secure scan architecture does not adopt the test key to protect chips, and thus the brute force attack based exhaustive search can not be applicable.

The timing influenced by the control logic insertion is evaluated by delay logic simulations. Top 10 critical paths are analyzed for AES circuits with standard scan design insertion and proposed secure architecture insertion, respectively. Experiment results show that, for either pipelined or iterative AES core, the top 10 critical paths before and after proposed secure architecture insertion keep unchanged. That is, the inserted control logic are not on the critical paths. Hence, the operating frequency of the circuit will not be degraded by the proposed secure scan architecture. Furthermore, the control logic cannot be corrupted by modifying the frequency of the main clock.

### 4.3. Overhead Analysis

In order to evaluate the area overhead, area simulations are also conducted on pipelined and iterative AES cores. The experimental results are given in Table 3. The areas are calculated as the number of equivalent two-input NAND gate. The columns labeled ‘Original’, ‘Standard’ and ‘Proposed’ show the areas of the original AES circuits, AES circuits with standard scan design insertion and AES circuits with proposed secure architecture insertion, respectively. The next-to-last column of Table 3 show the area penalty introduced by the proposed secure architecture in the form of equivalent logic gate. The last column shows the area overheads ratio compared with standard scan design.

The proposed technique is also compared with other countermeasures resisting scan attacks, including MKR [22], secure DFT [33], SOSD [37] and DOS [40] in Table 4. SOSD-64 and SOSD-128 refer to the SOSD design in [37] with 64-bit and 128-bit shift register (SR), respectively. DOS-10% refers to the DOS design in [40] with 10% permutation rate. As can be seen from the Table 4, the area penalty of the presented secure architecture is very low and almost negligible. The proposed secure scan architecture needs merely small amount of extra logic, which does not depend upon the circuit size and the number/length of scan chains.

Besides very low area penalty, this proposed architecture has no special requirements for system configuration and can be directly integrated into the scan design of crypto core. It does not involve modifying scan chain, and only needs to insert a flip-flop and a few logic gates in IP design. Extra control signals introduction Consequently, it has very small impact on IP design. There is also not setup time (extra clock cycles) before test. Thus, it will not increase the test time.

Table 5 gives comprehensive comparison with other secure scan techniques on performances except hardware overhead. Similar with the proposed technique, MKR [22], mode reset [31], smart controller [32] and secure DFT [33] are countermeasures based secure test control. All these countermeasures have connatural resistance to brute force and do not need extra test cycles. They also have common weakness that online testing is limited. This is the price of security improvement. Nevertheless, compared with other secure test control countermeasures the proposed technique can provide better protect for cryptographic chips with boundary scan design and requires less control logic insertion, as shown in second and fourth columns of Table 5. SOSD [37] is a scan data obfuscation countermeasure based on test key and lock. This countermeasure can thwart all known noninvasive attacks with the probability of brute force $2^{-64}$ or $2^{-128}$, which depends on the length of inserted shift register. For SOSD with 64/128-bit shift register, 64/128 clock cycles before testing are needed to deliver the test key into shift register. This countermeasure requires a certain amount of hardware insertion including test key loading controller and shift register and the modification of scan-enabling input in scan chains. It also limits the application of delay test based on LoC. DOS [40] perturbs test patterns and responses by inserting XOR gates into scan chains. It can resist all known noninvasive attacks with very small probability of brute force. However, it involves relatively complex hardware insertion and requires the scan chain modification. The scan chain encryption scheme [44] is not vulnerable to all known noninvasive attacks and can apply all kinds of tests, but it requires scan cipher insertion, which has a great impact on IP design. It also requires multiple clock cycles for pattern decryption before shifting in a test pattern. The scan chain scrambling scheme [48] divides the scan chain into multiple segments which are connected dynamically through a schan chain scrambler. When the test key is valid, the scan chain segments are connected in fixed order. Otherwise, they are connected in random order and thus scan output data is obfuscated. This technique can overcome all known noninvasive attacks with the probability of brute force $2^{-n}$ (*n* represents the length of test key). However, it needs complex control logic and also the scan chain modification.

As depicted in the table, the presented technique has the following merits: high security against external abnormal operation of scan-based test infrastructure, tiny impact on circuit design and no impact on test time.

## 5. Conclusions

The scan DFT methodology brings serious security risks to encryption IP core in sensor networks despite improving its test quality. In order to vanquish scan-based noninvasive attacks without sacrificing the test quality, this paper presents a secure scan test scheme for crypto chips. This secure test scheme offers the functional-mode shift disability mechanism, scan chain reset mechanism, and key isolation mechanism, thereby overcoming all potential scan attacks. Compared with other secure schemes, the area penalty of the presented architecture is very low and could almost be neglected. This is one of the most significant merits of the presented scheme.

## Figures and Tables

**Table 1:** The relation between {A1’, A2’ } and {Mode_sel, A1, A2}.

Mode_sel	A1 A2	A1’ A2’
1	00	11
1	01	01
1	10	10
1	11	11
0	00	00
0	01	01
0	10	10
0	11	11

**Table 2:** Test effectiveness results for standard scan design and proposed secure architecture.

AES	Standard Scan Design		Proposed Secure Architecture			
	Test Vectors	Fault Coverage	Test Vectors	Test Vectors Changed	Fault Coverage	Fault Coverage Changed
Pipelined	903	97.26%	910	+0.78%	97.23%	−0.03%
Iterative	608	97.90%	611	+0.49%	97.88%	−0.02%

**Table 3:** Synthesis results of original implementation, standard scan design and proposed secure architecture.

AES	Architecture			Area	Area Overhead
	Original	Standard	Proposed	Overhead	Ratio
Pipelined	205,934	217,720	217,804	84	0.039%
Iterative	25,052	29,032	29,053	21	0.072%

**Table 4:** Area overhead comparison of different secure schemes.

AES	Area Overhead Ratio						
	Proposed	MKR [22]	Secure	SOSD [37]		DOS [40]	
			DFT [33]	SOSD-64	SOSD-128	DOS-10%	DOS-30%
Pipelined	0.039%	0.15%	0.11%	0.18%	0.34%	0.85%	2.01%
Iterative	0.072%	1.32%	0.96%	1.52%	2.81%	-	-

**Table 5:** Comparison of different security scan schemes.

Scheme	Security		Impact on	Impact on Test	
	Vulnerability (*)	Probability of Brute Force	IP Design	Test Time	Test Application
Proposed	None	Brute force is inapplicable	A D flip-flop and a few logic gates insertion; no introduction of extra input signals	No extra cycles are needed	Online testing cannot be applied
MKR [22]	Test-mode-only attacks for boundary scan design	Brute force is inapplicable	Secure control circuit insertion; scan chain modification; extra control signals introduction	No extra cycles are needed	Online testing cannot be applied
Mode reset [31]	Test-mode-only attacks for boundary scan design	Brute force is inapplicable	System mode security manager, scan_enable integrity controller, reset controller and test controller insertion	No extra cycles are needed	Online testing cannot be applied
Smart controller [32]	Test-mode-only attacks for boundary scan design	Brute force is inapplicable	Smart controller and multiple multiplexers insertion	No extra cycles are needed	Online testing cannot be applied
Secure DFT [33]	Test-mode-only attacks for boundary scan design	Brute force is inapplicable	A small secure test controller and a few logic gates insertion	No extra cycles are needed	Online testing cannot be applied
SOSD-64 [37]	None	$2^{-64}$	Test key loading controller, shift register insertion; scan-enabling input modification in scan chains	64 clock cycles before testing	Delay test based on LoC cannot be applied
SOSD-128 [37]	None	$2^{-128}$	Test key loading controller, shift register insertion; scan-enabling input modification in scan chains	128 clock cycles before testing	Delay test based on LoC cannot be applied
DOS [40]	None	$2^{-k\lambda }$ (**)	LFSR, shadow chain and control unit insertion; scan chain modification	No extra cycles are needed	All the tests can be applied
Scan chain encryption [44]	None	$2^{-m}$ (***)	Scan cipher insertion at scan inputs and outputs	multiple clock cycles for pattern decryption	All the tests can be applied
Scan chain scrambling [48]	None	$2^{-n}$ (****)	Test configuration module, unpredictable number generator and multiple multiplexers insertion; scan chain modification	Immaterial	All the tests can be applied

Notes: (*) It’s the vulnerability to external abnormal operation of scan-based test infrastructure. Notes: (**) *k* and *λ* represent the number and length of parallel scan chains for the DOS scheme, respectively. Notes: (***) *m* represents the key length of scan cipher for the scan chain encryption scheme. Notes: (****) *n* represents the length of test key for the scan chain scrambling scheme.
